# The Use of a Dehydrated Amnion/Chorion Membrane Allograft in Patients Who Subsequently Undergo Reexploration after Posterior Lumbar Instrumentation

**DOI:** 10.1155/2015/501202

**Published:** 2015-01-13

**Authors:** Brian R. Subach, Anne G. Copay

**Affiliations:** ^1^Virginia Spine Institute & Spinal Research Foundation, 1831 Wiehle Avenue, Suite 200, Reston, VA 20190, USA; ^2^SPIRITT Research, 12977 North Forty Drive, Suite 200, St. Louis, MO 63141, USA

## Abstract

*Background Context*. Products that can reduce development of epidural fibrosis may reduce risk for ongoing pain associated with development of scar tissue and make subsequent epidural reexploration easier. *Purpose*. To evaluate the use of dehydrated human amnion/chorion membrane (dHACM) on the formation of soft tissue scarring in the epidural space. *Study Design*. Case series. *Patient Sample*. Five patients having transforaminal lumbar interbody lumbar fusion (TLIF) with posterior instrumentation and implantation of dHACM in the epidural space and subsequent epidural reexploration.* Outcome Measures*. Degree of scar tissue adjacent to the epidural space at reexploration. Intraoperative and postoperative complications related to dHACM and patient reported outcomes. *Methods*. The degree of scar tissue adjacent to the epidural space was assessed during the reexploration surgery. Patients' outcomes were collected using standard validated questionnaires. *Results*. Four of 5 cases had easily detachable tissue during epidural reexploration. Angiolipoma of 10% was noted in 1 case and 5% in 2 cases. Significant improvements in patient reported outcomes were observed. No intraoperative or postoperative complications occurred. *Conclusions.* Our findings suggest that dHACM implant during TLIF may have favorable effects on epidural fibrosis and is well tolerated. Further studies with larger cohorts are required to prove our results.

## 1. Background and Significance

Epidural fibrosis results from the surgical intervention in the epidural space. The scar tissue envelops the nerve roots and covers the exposed portion of the dura within the spinal canal. Although this process is normal and an expected result of healing of the surgical site or wound, actual formation of scar tissue and adhesions may lead to a less than optimal clinical outcome. Specifically, epidural fibrosis has been associated with the persistence or recurrence of back pain after discectomy [1–5]. Epidural fibrosis also increases the risk and difficulty associated with revision surgery. The surgical time is lengthened due to the difficulty of dissecting structures covered by scar tissue and the rate of complications such as dural tear, nerve damage, and bleeding is increased [6, 7].

A variety of methods have been used in an attempt to prevent and reduce epidural fibrosis. The most notable method may be the ADCON-L (Gliatech Inc., Cleveland, OH), a carbohydrate polymer gel designed to provide a mechanical barrier to inhibit fibroblast migration and adhesion. While the early results of the ADCON-L showed reduced scar tissue formation and improved outcomes [8, 9], the incidence of serious side effects [10–12] (cerebrospinal fluid leak, delayed wound healing, and pseudarthrosis) lead to the withdrawal of ADCON-L from the market.

Another antiadhesion gel (Oxiplex or Medishield, Fziomed Inc., San Luis Obispo, CA), composed of carboxymethylcellulose and polyethylene oxide, has been shown to improve some outcomes and reduce the rate of reoperation [13, 14]. The use of Oxiplex is limited by the fact that it is contraindicated in cases of dural tear and cerebrospinal fluid leakage. A possible alternative may be a synthetic hydrogel (DuraSeal Xact, Covidien, Mansfield, MA). DuraSeal is used as dural sealant and a pilot study reported on its additional antiadhesion properties. When applied as a thin layer to the nerve root and surrounding area, DuraSeal resulted in less scar tissue formation, better patient outcomes, and lower reoperation rate [15].

The use of human amniotic membrane based products has also been considered for the prevention of epidural fibrosis. Amniotic membrane, comprised of both amnion and chorion membranes, is metabolically active tissue which continually remodels the extracellular matrix through processes controlled by paracrine growth factors [16]. Amnion has 5 layers including epithelium, basement membrane, compact layer, fibroblast layer, and spongy layer. Chorion, composed of reticular layer, basement membrane, and trophoblast layer, is 3-4 times thicker than amnion. Human amniotic membrane contains growth factors which are known to stimulate epithelial cell migration and proliferation as well as many metabolic processes, including general protein and collagen synthesis, collagenase activity, and chemotaxis of fibroblasts and of smooth muscle cells [17]. Studies have shown that amniotic membrane has inherent properties which enhance the healing process. These properties include being immune privileged, reducing inflammation, and reducing scar tissue formation [17–20]. Human amniotic membrane also has antibacterial, hemostatic, and pain reduction properties, is self-signaling, and mediates tissue repair via the contained growth factors [20]. Amniotic membrane is used in a variety of applications such as conjunctival reconstruction, pterygium repair, the treatment of burns, ulcers, chronic wounds, and wound dehiscence [21]. An animal study has shown that amniotic membrane reduces postlaminectomy epidural adhesions [18].

Dehydrated human amnion/chorion membrane (dHACM) is a dehydrated human allograft comprised of laminated amnion and chorion membranes derived from donated human placentas according to the American Association of Tissue Banks (AATB) standards and is considered a tissue product under Section 361 of the Public Health Service Act. PURION processed dHACM has been shown to retain the growth factors in natural amniotic membrane including PDGF-AA, PDGF-BB, bFGF, TGF-*β*1, EGF, VEGF, and PlGF [22]. In addition to growth factors, cytokines including anti-inflammatory interleukins (IL-1ra, IL-4, and IL-10) and the TIMPs (TIMP-1, TIMP-2, and TIMP-4) which help regulate the matrix metalloproteinase (MMP) activity are also present in dHACM [22].

Our primary objective was to evaluate the use of dehydrated human amnion/chorion membrane (dHACM) allograft (AmnioFix, MiMedx, Marietta, GA) and the formation of soft tissue scarring in the epidural space, in patients having transforaminal lumbar interbody lumbar fusion (TLIF) with posterior instrumentation and subsequent reexploration with instrumentation removal. Secondary outcomes included intraoperative and postoperative complications related to dHACM and patient outcomes relative to disability, pain, and functional health.

## 2. Methods

### 2.1. Study Population

The study design, protocol, and consent forms (clinical trial number: NCT01357187) were approved by the local Institutional Review Board (HCA Reston Hospital, Reston, Virginia). All participants gave full informed consent to participate in the study. Patients were eligible to receive dHACM implantation if they had no history of previous spinal surgery and were scheduled for transforaminal lumbar interbody fusion (TLIF) with posterior instrumentation. Included were those patients that underwent a secondary surgery to remove the posterior segmental instrumentation and required exploration of the appropriate nerve roots after having achieved solid bony fusion.

### 2.2. Surgical Procedures

#### 2.2.1. Transforaminal Lumbar Interbody Fusion (TLIF)

Patients underwent standard TLIF with posterior pedicle screw fixation. After the insertion of the interbody fusion device, bone graft material, and posterior stabilization devices, the dHACM (AmnioFix, MiMedx, Marietta, GA) was oriented with the appropriate side up, cut to fit the dura exposed by the decompression, and placed in the epidural space. Meticulous hemostasis was achieved and a hemovac drain placed prior to routine closure.

#### 2.2.2. Instrumentation Removal

After radiological studies had been performed to document adequate fusion, as evidenced by bridging bone within the interbody space and adjacent intertransverse region, the patient was scheduled for instrumentation removal with exploration of the epidural space. After exposure, the hardware was removed and the pedicles reconstructed with crushed cancellous allograft. The ease of dissection/extent of adhesions in the epidural space was determined by the surgeon and a piece of tissue adjacent to the epidural space was sent for histopathologic analysis.

### 2.3. Assessment of Scarring

During the instrumentation removal surgery, the spine surgeon assessed the degree of scar formation adjacent to the epidural space using a 4-level scoring system ranging from “no adhesion” to “sharp dissection required” (Table 1).

### 2.4. Histological Analysis

During the instrumentation removal surgery, a small sample of the scar tissue adjacent to the epidural space was collected for histological analysis. A 1-cm^2^ section of the lumbar tissue was removed with a small scalpel blade, clearly marking the surface adjacent to the dura. The tissue sample was treated with H & E staining. The slides were evaluated for the presence and extent of scar tissue (mm of fibrosis and percent of fat infiltration).

### 2.5. Complications and Patients' Outcomes

Intraoperative and postoperative complications were recorded. Patients were evaluated at three weeks, three months, six months, and twelve months after the TLIF surgery and again after the instrumentation removal surgery. Patients' outcomes were collected using standard validated questionnaires including Oswestry Disability Index (ODI), Numerical Rating Pain Scales, and SF-36. The average change in patient outcomes was assessed with repeated-measures analysis (SPSS Inc, Chicago, IL) with alpha = 0.05.

## 3. Results

**Figure 1:**
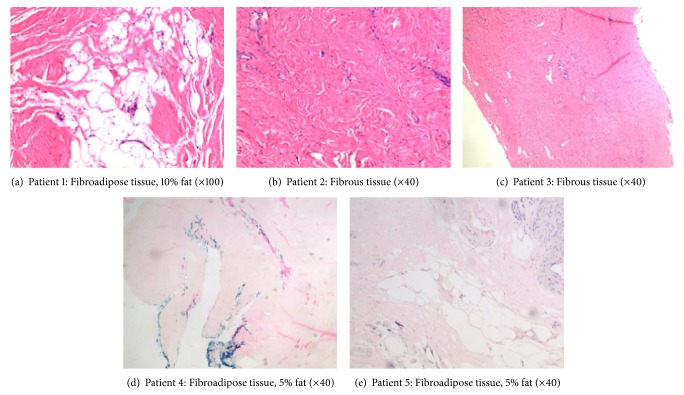
Histologic overview (H & E, original magnification ×100 or ×40).

During the study period a total of 5 patients (males, average age 61 years) without history of prior spinal surgery underwent TLIF with dHACM and subsequent reexploration. Patient diagnosis, levels of TLIF, adhesion score assessed at reexploration, and histological analysis of fibrosis and fat infiltration are reported in Table 2. Four of 5 cases had easily detachable tissue during instrumentation removal. The histological analysis showed minimal fibrosis and fat infiltration in most cases (Table 2 and Figure 1).

### 3.1. Complications

There were no intraoperative complications, specifically no durotomy/cerebrospinal fluid leaks.

Similarly, there were no postoperative complications, specifically no wound infections/dehiscence.

### 3.2. Patient Outcomes

**Figure 2:**
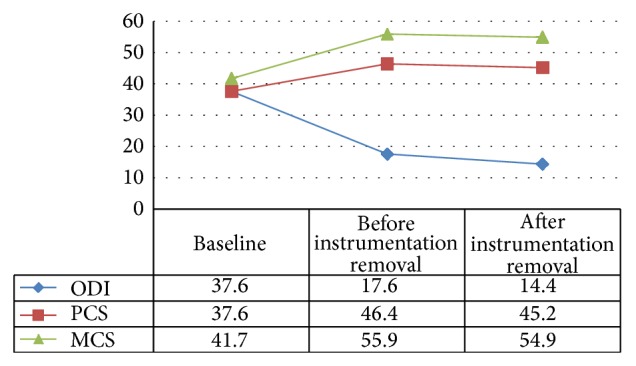
Sample averages for the Oswestry Disability Index (ODI), the Physical Component Summary (PCS), and the Mental Component Summary (MCS) of the Medical Outcomes Study Questionnaire Short Form 36. ODI: *P* = 0.0032 ; MCS: *P* = 0.0239.

**Figure 3:**
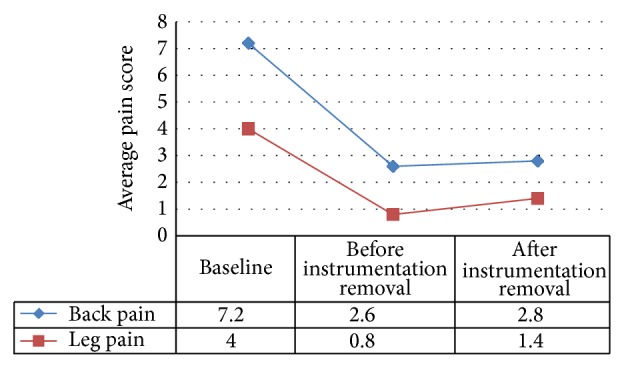
Sample averages for back pain and leg pain.  Back pain: *P* = 0.007.

Individual patient outcomes are reported in Table 3 and the averages for the patient sample are reported in Figures 2 and 3. The Oswestry Disability Index (ODI) measures disability; a lower ODI score indicates improvement. Similarly, a lower score on the numerical rating scale for back pain and leg pain indicates a lower pain level. The Physical Component Summary (PCS) and the Mental Component Summary (MCS) of the Medical Outcomes Study Questionnaire Short Form 36 are an indication of physical health and mental health, respectively. A higher score indicates improvement for PCS and MCS. On average, patients show a significant improvement in disability (ODI) and mental well-being (MCS).

While there is also an improvement in physical well-being (PCS), it is not statistically significant (Figure 2).

Both back and leg pain decreased on average. The decrease was significant for back pain but not leg pain (Figure 3).

Patients exhibited individual variation in their responses to treatment: half the patients reported minimal to no disability and pain while half the patients improved but experienced persistent pain and disability. Patient 2 underwent a sacroiliac joint fusion to further help with back pain. Patient 4 underwent an unrelated cervical fusion for cervical myelopathy.

## 4. Discussion

Supplemental instrumentation is widely used in spinal fusion surgery. Biomechanical studies have demonstrated that supplemental instrumentation enhances the stability of the target motion segment [23]. A subsequent operation to remove the instrumentation may be indicated due to infection or for patients who continue to experience pain due to myofascial irritation, prominent hardware, sacroiliac joint disease, or persistent or recurrent radicular symptoms [24–27].

This pilot study investigated the use of a dHACM barrier in reducing epidural fibrosis and facilitating dissection in revision spinal surgery. Although the small sample size of the study limits the ability to generalize our results, we believe the use of a dHACM barrier was clearly useful in limiting epidural fibrosis and promoting dissection in revision spinal surgery. Furthermore, the dHACM did not lead to any adverse events such as infection or spinal fluid leak.

Compared to other barriers that we have utilized, the dHACM performed better in revision surgery. In the past, we have used ADCON-L gel (Gliatech, Cleveland, OH, USA) but no longer utilize the product given the reports in the literature of associated radiculitis and spinal fluid leak. We have also used DuraSeal Xact (Covidien, Mansfield, MA) but contrary to Fransen study [15], we did not see a significant reduction in scar tissue formation. We have also used the GORE-TEX barrier, Preclude Spinal Membrane (Gore, Flagstaff, AZ), but found it difficult to manipulate.

## 5. Conclusion

Although this study is too small to properly evaluate the effectiveness of dHACM in preventing epidural fibrosis and facilitating dissection in revision spinal surgery, it clearly shows promise in achieving these goals. These results and the impact of the dHACM barrier on patient outcomes need to be confirmed with larger studies with longer term follow-up, before more definitive conclusions may be drawn.

## Figures and Tables

**Table 1:** Scoring scheme for the presence of adhesions and quality of the dissection plane.

Score	Description
1	**Separates with no adhesion**—applicable tissues can be easily separated from the study site without the use of surgical dissection

2	**Easily detachable**—applicable tissues can be safely separated from the study site with minimal use of blunt surgical instruments to overcome light adhesion

3	**Dissection required**—applicable tissues can be safely separated from the study site while using blunt surgical tools to overcome moderate adhesion

4	**Sharp dissection required**—applicable tissues cannot be separated from the study site without risk of damage as the use of sharp surgical tools is required to overcome tenacious adhesion

**Table 2:** Adhesion score, fibrosis, fat infiltration, and length of surgery.

Patient	Diagnosis	TLIF	Adhesion score	Fibrosis (mm)	% fat infiltration	Surgery time (min)
1	Spondylolisthesis	L4-L5	4	2.5	10	*101 *
2	Stenosis	L4-L5	2	5	0	*135 *
3	Spondylolisthesis	L3-L5	2	2.5	0	*119 *
4	Stenosis	L3-L5	2	6	5	*116 *
5	Spondylosis	L4-S1	2	2.5	5	* 77 *

Adhesion score: 4 = sharp dissection required; 2 = easily detachable.

**Table 3:** Patient outcomes.

Patient	Before TLIF					Before instrumentation removal					After instrumentation removal				
	ODI	Back pain	Leg pain	PCS	MCS	ODI	Back pain	Leg pain	PCS	MCS	ODI	Back pain	Leg pain	PCS	MCS
1	62	7	4	42.0	22.0	54	3	1	40.4	35.2	33	4	4	30.6	30.4
2	32	8	0	41.6	36.9	20	6	0	44.3	61.7	24	5	0	37.0	63.2
3	32	7	5	29.5	52.6	6	2	2	47.7	60.2	2	2	3	47.8	61.1
4	22	6	3	32.2	50.9	8	1	0	52.7	61.1	0	3	0	45.5	61.1
5	40	8	8	42.5	46.1	0	1	1	46.9	61.5	0	0	0	55.8	58.9

Average	37.6	7.2	4.0	37.6	41.7	17.6	2.6	0.8	46.4	55.9	14.4	2.8	1.4	45.2	54.9
